# The Quality of Family Medicine Team Conferences Through the Lens of a Director: An Autoethnography

**DOI:** 10.7759/cureus.64173

**Published:** 2024-07-09

**Authors:** Ryuichi Ohta, Chiaki Sano

**Affiliations:** 1 Community Care, Unnan City Hospital, Unnan, JPN; 2 Community Medicine Management, Shimane University Faculty of Medicine, Izumo, JPN

**Keywords:** general medicine, rural, patient care management, medical education, interprofessional relations, team-based care, family practice, rural health services

## Abstract

Introduction

In rural medical settings, team conferences are essential for effective patient care, especially given the challenges of limited resources and personnel. These conferences promote collaborative discussions on patient management and serve as vital educational sessions. This study explores the dynamics and efficacy of team conferences in the family medicine department of a rural hospital to optimize patient care and educational outcomes.

Methods

This qualitative study used autoethnography at Unnan City Hospital, Unnan, Japan. Data collection included semi-structured interviews, direct observation, reflective field notes, and informal conversations with medical students, junior residents, and general medicine trainees. The focus was on conference interactions, educational content, and operational challenges. Data analysis involved coding and theming, with ongoing discussions among researchers and participants to refine findings.

Results

Three key themes emerged. First, patient outcomes suffered from a lack of professional awareness. Second, mutual understanding and individual autonomy enhanced team quality. Third, team healthcare quality improved through diverse and inclusive learning experiences. Effective facilitation, structured time management, and integrating practical bedside learning with theoretical discussions were crucial for optimizing team conferences. Psychological safety, respect for individual differences, and maintaining motivation were essential for productive team interactions.

Conclusion

The study highlights the importance of effective facilitation, time management, and integrating practical and theoretical learning in enhancing team conferences in rural medical settings. Psychological safety and mutual respect are vital for fostering a collaborative and motivated team environment. Addressing these factors can improve patient care and educational experiences. Future research should include diverse settings and quantitative measures to validate and refine these insights, enhancing team conferences in rural healthcare environments.

## Introduction

In the multifaceted landscape of medical care management, team conferences are pivotal in ensuring effective patient care [1]. These conferences, particularly those convened among physicians within medical institutions, serve as a critical forum for the collaborative discussion of patient management strategies and healthcare delivery [2]. They are integral for sharing information about patients’ conditions and ensuring the continuity of care, especially when individual physicians are unavailable [3].

Team conferences are not monolithic; they vary widely in context, methodology, and purpose. Depending on the healthcare setting, these meetings can focus on the sustainability of medical operations or serve as educational platforms for residents and junior physicians [4]. Medical teachers can teach concrete medical essence and patient management in team conferences [5]. However, the efficacy of these conferences can be hindered by challenges such as effective time management and sustaining participant motivation [6].

This study focuses on the unique dynamics of team conferences within rural medical settings, where family-physician interactions are both frequent and critical due to the scarcity of healthcare professionals [7]. Medical professionals have multiple tasks and have few opportunities to share their patients’ information and educate each other in teams [8]. In these environments, team discussions take on heightened significance. They are not only necessary for managing the immediate medical needs of patients but also serve as critical educational sessions that address the biopsychosocial aspects of patient care, involving a wide range of healthcare providers, from medical students to family physicians [9]. Effective management of team conferences can be an essential platform for medical management and education in rural contexts.

Particularly in rural family medicine departments, where the spectrum of patient issues is broad and complex, the role of team conferences extends beyond routine patient care; they are vital for continuous medical education and professional development [10,11]. This study aims to explore and clarify team conferences’ operational challenges and efficacy in these settings. It seeks to understand how they can be optimized to improve patient care and educational outcomes. By doing so, it intends to contribute valuable insights into the administration and enhancement of team conferences in rural healthcare environments, ensuring they are both effective in practice and beneficial in educational scope.

## Materials and methods

Research design

This study employed a qualitative research approach using autoethnography to examine the operational challenges and efficacy of team conferences in the family medicine department of a rural community hospital through the lens of a director of a rural family medicine department and critical reflection with team members [12]. Autoethnography allows for an in-depth exploration of practices from an insider’s perspective, providing nuanced insights into the healthcare team’s daily workings and interpersonal dynamics [13]. This method is particularly suited for studying complex social interactions and organizational cultures within healthcare settings. In this research design, to overcome the limitation, data collection used one-on-one semi-structured interviews, observation of the researcher’s feelings and participants’ interactions, field notes regarding team conferences, and reflections with participants about team conferences for member checking [12].

Setting

The study was conducted at the Unnan City Hospital, Unnan, Japan. The hospital has 281 care beds: 160 for acute care, 43 for comprehensive care, 30 for rehabilitation, and 48 for chronic care. It provides general medicine training to medical residents. Under this curriculum, medical residents experience multiple clinical scenarios in treating their patients: inpatient, outpatient, home, and community care. The General Medicine Department mainly manages its educational curriculum. The department promotes interprofessional collaboration with dentists, pharmacists, therapists, nurses, and nutritionists. Interprofessional collaboration in managing older inpatients has been shown to reduce readmission rates, which can drive interprofessional education among the residents [14,15].

Unnan City Hospital offers community-based medical education (CBME) to students and residents from various institutions and provides a diverse range of clinical exposure in different care settings [16]. Before joining, students and residents typically undergo a month’s training in rural general medicine at other facilities. However, students observe under medical teachers, while residents can independently assess patients but must consult before prescribing or ordering tests [17]. Unnan City Hospital hosts 40-50 trainees annually in its Department of General Medicine.

Participants

The research participants comprised general medicine trainees from the Family Medicine Department of the Unnan City Hospital. Three medical teachers specialized in family medicine at the Unnan City Hospital during the study period. Before joining the Unnan City Hospital, the medical residents had received rural family medicine education at medical universities and tertiary hospitals. They had also trained in family medicine at rural hospitals for a year with medical teachers and other family medicine residents. One resident started training in the curriculum in 2018 and 2019, and three in 2020, 2021, and 2022. In 2022, 2023, and 2024, these 13 physicians (three family physicians and nine family medicine residents) were part of the General Medicine Department.

This study included medical students and junior residents. Between April 2021 and March 2023, 69 trainees (53 medical students and 16 junior residents) undertook the CBME curriculum at Unnan City Hospital. This curriculum aims to instill essential competencies in general medicine as recognized by Japan. Those motivated toward general medicine were informed about the study using purposive sampling. In total, 26 medical students, four junior residents, 12 general medicine residents, and teachers consented to participate [9].

Educational content for integration and psychological safety

The department provides medical residency training through an educational curriculum aligned with the Japanese Primary Care Association’s Board of Family Medicine, developed following the World Standard of Education of Family Medicine. This curriculum can simultaneously educate up to three residents, reflecting the hospital’s educational capacity. The residency program includes three instructors and exposes residents to various clinical scenarios. Residents manage common diseases in inpatient and outpatient settings at Unnan City Hospital in their first year. The following year, they spent six months at a rural clinic (Kakeya Clinic) to gain experience in home care and community-oriented primary care. To broaden their scope in internal medicine, pediatrics, and emergency medicine, residents also spend 18 months at a general or community teaching hospital. Each clinical setting includes guidance from a medical teacher [9].

We implemented near-peer learning methods by defining each team member’s role [18]. As part of a process improvement program, we adopted the shared reading approach to improve psychological safety and bridge the generation gap in our department. First- and second-year senior residents were responsible for patient management and educating medical students and junior residents. They were also instructed to accompany these junior colleagues during outpatient and ward duties as much as possible and reflect on their learning experiences, ensuring psychological safety. Third-year residents provided feedback to first- and second-year residents and managed the overall supervision of senior residents. Additionally, third-year residents served as liaisons between medical teachers and second-year residents. The medical teachers oversaw the entire residency program while discussing patient management with the third-year residents.

The shared reading approach was centered around medical topics based on participants’ interests. Initially, the first researcher (RO) identified topics by discussing the participants’ clinical questions and learning difficulties in the educational program [19]. After selecting a topic, an appropriate book was chosen and shared with the participants. Each shared reading group consisted of three to four members, and the book was divided into sections for daily reading. Members shared their learning points in a closed social media group, where others could comment based on their learning experiences.

The topics and books used in shared reading were related to family medicine and chronic medical conditions, such as concepts of family medicine, cardiovascular disease, fever of unknown origins, general internal medicine management, and palliative care. Only Japanese texts were used, as Japanese medical students often prefer reading in their native language [19]. The knowledge gained through shared reading was presented at the Department of Family Medicine conferences at Unnan City Hospital, allowing all team members to expand their understanding. Participants could join or leave the shared reading groups based on their availability [20].

Team conferences

Every morning, from 7:30 to 8:30 am, from Monday to Friday, the Department of Family Medicine holds regular team conferences to review and discuss patients whose conditions are deteriorating or concern chief physicians. The review and discussion were facilitated by family medicine residents in the department. The facilitators changed each day, and the duration was about 30 minutes. The medical educators listen to the presentations of family medicine residents and derive their difficulty in management, suggesting the possible management of their patients from practical and educational perspectives.

In addition, the conference includes educational sessions for family medicine residents regarding what they have learned from previous conferences. After each conference, medical residents discuss with their family medicine educators and find learning points from their cases. They decide on one specific learning point, which the resident presents to the other conference members. In the next week, the residents make a brief presentation for about five to 10 minutes and share the presentation with the educators for feedback. In the next conference, one resident presents their learning contents and discusses with the other members the improvement of quality of care in the Department of Family Medicine.

Data collection

Data was collected by the primary researcher, embedded in the setting as a family medicine educator (RO). This dual role of researcher and practitioner provided a unique vantage point, facilitating access to authentic interactions and experiences. The data was gathered through direct participation, observation, reflective field notes, and one-on-one semi-structured interviews [12,13].

Observations

RO participated in morning team conferences and discussed family residents’ cases daily. In the process, RO observed the participants’ discussion contents and interactions with the members. In addition, RO observed their collaboration in patient care in outpatient and inpatient departments in the hospital. RO focused on systematic observation of team conferences, communication patterns, decision-making processes, and the integration of educational content in the Department of Family Medicine patient care and the changes in the relationships of members and discussion contents [12,13].

Reflective Field Notes

RO maintained detailed notes on personal reflections, interactions, and incidents, highlighting the team’s operational dynamics and educational interactions in team conferences and outpatient and inpatient departments. For example, RO took notes while listening to family medicine teachers, residents, junior residents, and medical students in team conferences. The team conferences’ discussions were not recorded because of the personal information of patients and families. RO took notes mainly regarding how the participants discussed topics with eye contact, gestures, and conflict management among members [12,13].

Informal Conversations

Casual, unstructured discussions with participants to gather spontaneous insights and perspectives about the team conferences were performed when RO had questions about the participants’ feelings about some conflicts. RO tried to investigate their feelings about the conflicts and learn from them open-mindedly [12,13].

One-on-One Interview

RO conducted monthly one-on-one interviews with the participants to investigate their perceptions regarding team meetings and revising points. Each session lasted 30 minutes, and each participant was interviewed in a conference room each month. RO reviewed relevant documents in the interviews, including meeting agendas, minutes, and training materials used during the conferences.

Data analysis

An autoethnographic approach was employed in this study to deeply explore the experiences and insights of the participants [12,13]. This method involved multiple stages of data collection and analysis. Initially, the primary researcher (RO) meticulously reviewed the field notes, conducted semi-structured in-depth interviews, and engaged in discussions with the research participants. The content from these activities was systematically coded, with RO developing codebooks through repeated reviews of the field notes to ensure initial coding reliability. The analysis predominantly utilized process and concept coding methods to identify key themes and patterns [12]. Following the initial coding, RO undertook a detailed process of induction, merging, deleting, and refining codes. This iterative process involved moving back and forth between the research data and the initial coding, allowing for the formation of more refined concepts. The second coding phase concentrated on grouping these tentative concepts and developing tentative themes, further refining the codes, concepts, and themes in an ongoing, iterative manner. RO and the research participants engaged in continuous discussions about the emerging concepts and themes to enhance the study’s validity through triangulation. The content of each semi-structured interview was analyzed iteratively, with RO refining the analysis after each interview. This continuous analysis allowed for the dynamic development of insights and themes. In the final stage, RO collaborated with CS to address and finalize the themes, ensuring that the conclusions were robust and reflected the data collected. The study comprehensively understood the research subjects’ experiences and perspectives through this rigorous and iterative process.

Reflexivity

This study’s results were cocreated by the researchers and participants through interactions. The research team members possessed diverse expertise and perspectives on rural medical education. RO, a family physician and medical teacher, graduated with a master’s degree in medical education and family medicine and has experience in working, education, and research in rural contexts. CS, a medical educator and professor at a medical university, had graduated from a medical university and specialized in community health care management and education. To prevent biases, the research team cautiously discussed the findings from individual data analyses. We explored alternative viewpoints while determining the meaning of the data.

Ethical consideration

The Unnan City Hospital Clinical Ethics Committee approved the study protocol (20240002).

## Results

The results of this study reveal critical insights into the operational dynamics and educational efficacy of team conferences in a rural family medicine department. Key themes emerged around the need for continuity in patient outcomes due to a lack of professional awareness, the importance of mutual understanding and individual autonomy to enhance team quality, and the improvement of team healthcare quality through diverse, inclusive learning. Participants highlighted the significance of effective facilitation, time management, and integrating practical bedside learning with theoretical discussions. The study also emphasized the necessity of psychological safety, respecting individual differences, and maintaining motivation and cohesion within the team. These findings underscore the complex interplay between team interactions, educational methods, and patient care in rural medical settings (Table 1).

**Table 1:** Themes and concepts of critical insights into the operational dynamics and educational efficacy of team conferences in a rural family medicine department

Theme	Concepts
Continuity in Patient Outcomes Due to a Lack of Professional Awareness	Attitudes Toward Educator Participation in Conferences
	Consideration of Time Constraints
	Concerns Over Theoretical Discussions
	Disconnection From Bedside Learning
Mutual Understanding and Individual Autonomy to Enhance Team Quality	Promoting Overall Growth
	Deepening Understanding of Psychological Safety
	Activating Discussions by Valuing Differences
	Enhancing Discussions Through Active Speaking
	Building Understanding Through Regular Gatherings
Improving Team Healthcare Quality Through Diverse, Inclusive Learning	Strengthening Team Unity
	Ensuring Comprehensive Learning for All Participants
	Balancing Individual and General Education
	Sustaining Participation and Motivation
	Integrating Discussions With Bedside Learning
	Respecting Diversity and Individual Volition

**Figure 1:**
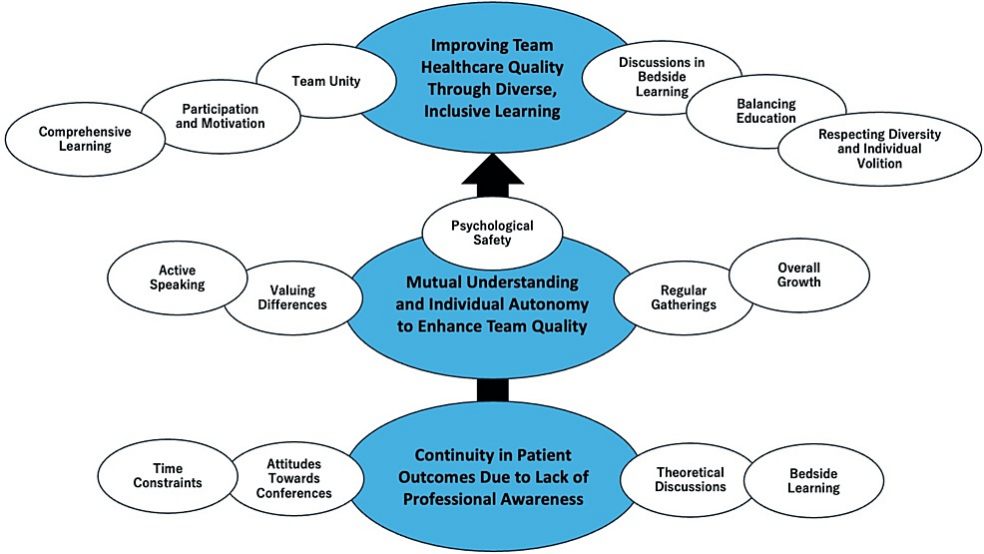
Conceptual framework of critical insights into the operational dynamics and educational efficacy of team conferences in a rural family medicine department Image credit: Ryuichi Ohta

Figure 1 shows the conceptual framework of the analysis.

Theme: Continuity in Patient Outcomes Due to a Lack of Professional Awareness

Attitudes Toward Educator Participation in Conferences

Participants emphasized the significance of the facilitator’s role in team conferences. One participant noted, “The presence and active contribution of team leaders are crucial. Their engagement can significantly encourage others to participate more proactively.” (Participant 2, family medicine resident) Another participant highlighted the importance of the facilitator’s role in the Japanese context, stating, “Considering the cultural context of Japan, effective facilitation is essential. It helps guide the entire team and ensures that discussions are productive and inclusive.” (Participant 6, medical student) The participants hoped that educators and leaders should facilitate the initial part of the team conferences to mitigate participants’ opportunities to participate in discussion in cases. These insights underscore facilitators’ need to actively lead and engage in team conferences to foster a collaborative and motivated environment.

Reflection of Time Constraints

The importance of time management was frequently discussed. One participant remarked, “Our discussions often become prolonged, and it’s crucial to establish strict time limits to ensure meetings conclude promptly.” (Participant 1, family medicine resident) Another participant echoed this sentiment, emphasizing, “We need to cultivate an awareness of mutual time usage. Everyone should be conscious that we are using each other’s time, and it is essential to manage it effectively.” (Participant 7, family medicine resident) In rural hospitals, family physicians and residents have various jobs to do in a day. Participants considered that although team conferences were important, time management in a day should be respected for effective professional work. These statements highlight the participants’ recognition of the need for structured time management to maintain the efficiency and productivity of team conferences.

Concerns Over Theoretical Discussions

There was apprehension among participants about discussions becoming overly theoretical and disconnected from practical applications. One participant expressed concern, saying, “Our discussions sometimes become too theoretical, which makes it hard to apply the insights to actual patient care.” (Participant 8, junior resident) Another participant emphasized the need for practical relevance, stating, “Continuous information sharing and joint patient assessments are crucial. They help ensure that our discussions are grounded in real patient cases and not just theoretical concepts.” (Participant 4, family medicine resident) Although discussing theoretical diseases was interesting for participants, their inability to apply them to clinical situations demotivated them regarding team conferences. These concerns underscore the importance of maintaining a balance between theoretical discussions and practical applications to enhance the effectiveness of team conferences.

Disconnection From Bedside Learning

Participants voiced concerns that team meetings might detract from bedside learning. One participant noted, “There’s a risk that our focus on verbal discussions during meetings could reduce the time we spend on direct patient care, which is critical for improving patient management.” (Participant 8, family medicine resident) Another participant echoed this sentiment, stating, “I’m worried that too much emphasis on discussion might lead to less bedside learning, which is where we gain the most practical experience.” (Participant 5, medical student) The participants experienced the discrepancy between team discussions and concrete patient critical courses. In reality, they had some difficulties with patient management because of the distorted suggestions from team discussions. These concerns highlight the need to balance team meetings with sufficient bedside learning to ensure comprehensive and effective patient care. They considered that practical bedside discussion can improve the quality of team discussion and patient care.

Theme: Mutual Understanding and Individual Autonomy to Enhance Team Quality

Promoting Overall Growth

Participants stressed the importance of providing individualized feedback to each learner while aiming to enhance the overall quality of the team. One participant remarked, “It’s crucial to give personalized feedback to each member, but we should also use these opportunities to improve the team.” (Participant 1, family medicine resident) Another participant suggested, “Team discussions can serve as platforms where we address and resolve broader questions that affect everyone. This way, individual learning contributes to the team’s collective growth.” (Participant 10, family medicine resident) Through the team conferences, the participants hoped to improve not only their abilities in clinical management but also the whole ability of the team to generate better patient outcomes. These insights highlight the dual focus on personal development and team enhancement as essential for practical team conferences.

Deepening Understanding of Psychological Safety

Many participants believed that psychological safety, where team members felt free to express their opinions, was crucial for effective and smooth team discussions. One participant emphasized, “Psychological safety is essential. Everyone should feel comfortable expressing their opinions without fear of judgment.” (Participant 11, medical teacher) Another participant noted, “Excessive attention to hierarchy and worrying too much about others’ feelings can hinder productive discussions. We need an environment where everyone feels safe to speak up.” (Participant 13, medical resident) Through the consecutive team conferences, the participants realized psychological safety and environments in which each participant could openly say what they considered to other members. Such an environment was generated by each participant’s effort to accept other participants’ ideas. These observations underscore the importance of fostering a psychologically safe space to ensure that team discussions are both practical and inclusive.

Activating Discussions by Valuing Differences

Participants valued respecting individual differences within the team. One participant remarked, “It’s important to respect each person’s unique perspective. This diversity enriches our discussions and leads to better outcomes.” (Participant 9, family medicine resident) Another participant emphasized the balance between open dialogue and time management, stating, “While fostering free discussions is crucial, we also need to adhere to time constraints to ensure our meetings are productive.” (Participant 16, medical student) Emphasizing individual interests and maintaining respect for differences were seen as vital for enriching team discussions, as one participant noted, “By valuing each other’s interests and perspectives, we can have more meaningful and effective conversations.” (Participant 14, family medicine resident) Respecting differences in perception and thoughts about clinical findings and social conditions of patients deepened the discussion in team conferences. The discussion became more sophisticated owing to respect for each other’s work and learning. These insights highlight the importance of inclusivity and structured dialogue in team meetings.

Enhancing Discussions Through Active Speaking

There were concerns about the tendency of participants to defer to each other overly. One participant mentioned, “Sometimes, we defer to each other too much, which can stall the conversation.” (Participant 20, family medicine resident) Another participant highlighted the role of educators in these situations, stating, “During moments of sparse dialogue, educators must step in and actively facilitate the discussion.” (Participant 1, family medicine resident) They believed that educators should invigorate the conversations to maintain engagement and productivity. One participant said, “Japanese culture can affect our discussion style. The members can be inward. Active speaking from educators can help keep the discussions lively and ensure that everyone’s voice is heard.” (Participant 21, medical teacher) They considered that Japanese culture could typically affect their discussion, which could impinge on it. These comments emphasize the need for proactive facilitation to enhance the effectiveness of team discussions.

Building Understanding Through Regular Gatherings

Participants highlighted the significance of daily team conferences in fostering a sense of unity. One participant noted, “Having regular daily meetings helps build a strong sense of team unity.” (Participant 17, family medicine resident) Another participant emphasized the role of these gatherings in enhancing mutual understanding, stating, “Regular interactions are crucial for deepening our understanding of each other’s thought processes and approaches.” (Participant 19, medical student) They believed that these consistent meetings contributed to better teamwork and collaboration. As one participant put it, “Daily team conferences allow us to share our perspectives and align our strategies, which is essential for effective patient care.” (Participant 2, family medicine resident) Constant team conferences facilitated the team members to understand each other and build their relationships effectively. These insights underscore the importance of regular, consistent gatherings in building a cohesive and collaborative team environment.

Theme: Improving Team Healthcare Quality Through Diverse, Inclusive Learning

Strengthening Team Unity

Participants believed that daily discussions during team conferences helped build team cohesion. One participant noted, “Daily discussions are vital for strengthening our team unity. They help us stay connected and work together more effectively.” (Participant 11, medical teacher) Another participant emphasized the practical benefits of these interactions, stating, “Regular meetings allow us to align on treatment directions and plan for the future collectively.” (Participant 10, family medicine resident) They felt that these consistent interactions were crucial for maintaining a unified approach to patient care. As one participant summarized, “By meeting daily, we ensure that everyone is on the same page, which is essential for delivering cohesive and coordinated care.” (Participant 1, family medicine resident) The improved unity of the team contributed to the team members’ satisfaction and effective patient care through collaboration. These observations highlight the role of regular discussions in fostering a cohesive and aligned team.

Ensuring Comprehensive Learning for All Participants

Participants sought personal growth through team conferences. One participant emphasized, “I want these conferences to help me grow, not just serve as a platform for sharing information.” (Participant 24, junior resident) Another participant shared a similar sentiment, stating, “It’s important that our participation in these meetings contributes to our personal and professional development.” (Participant 7, family medicine resident) There was a strong desire for the conferences to transcend simple information exchanges. As one participant put it, “We need these gatherings to be more than just passing along information; they should be opportunities for us to develop and advance in our careers.” (Participant 8, family medicine resident) Another participant echoed this, noting, “The real value of these conferences lies in their ability to foster our growth and make us better at what we do.” (Participant 10, family medicine resident) The team conferences were considered opportunities for continuous team and personal advancements. These quotes highlight the participants’ aspiration for team conferences to be enriching experiences facilitating comprehensive learning and development.

Balancing Individual and General Education

Participants sought personal growth through team conferences. One participant expressed, “Our participation in these conferences must contribute to our development, not just information sharing.” (Participant 13, junior resident) Another participant emphasized the need for these meetings to go beyond simple exchanges of information, stating, “We strongly desire for the conferences to serve as platforms for both personal and professional growth.” (Participant 5, medical student) They felt that these gatherings should be enriching and developmental experiences. As one participant put it, “These conferences should help us grow as individuals and professionals, making the time we spend in them truly valuable, and eventually leading the quality of patient management.” (Participant 3, medical resident) The participants respected personal growth through team conferences and recognized the balance between personal and team growth for better care of patients. These insights highlight the participants’ aspiration for team conferences to be comprehensive learning experiences that foster growth and development.

Sustaining Participation and Motivation

Participants recognized the importance of maintaining motivation for continuous participation. One participant remarked, “Keeping everyone motivated is crucial for ensuring ongoing participation in our team conferences.” (Participant 11, medical teacher) Another participant highlighted specific challenges, noting, “We face issues with varying levels of patient familiarity among team members, which can affect the quality of discussions.” (Participant 10, family medicine resident) Additionally, there were concerns about the impact of punctuality on team dynamics, as one participant mentioned, “Negative feelings toward latecomers can disrupt the flow and morale of the group.” (Participant 2, family medicine resident) They believed sustaining participation required maintaining a certain level of motivation among all participants. One participant said, “To keep everyone engaged, we need to find ways to ensure that everyone remains motivated and feels valued in these meetings.” (Participant 21, medical teacher) The participants considered that for the sustainability of team conferences, the professionalism of each member was essential, with respect for other participants. These insights emphasize the need for strategies to sustain motivation and participation in team conferences.

Integrating Discussions With Bedside Learning

Participants expressed a desire to verify the contents of team discussions at the bedside with mentors and peers. One participant explained, “It’s important for us to take what we’ve discussed in team meetings and apply it at the bedside with the guidance of our mentors and peers.” (Participant 6, junior resident) Another participant emphasized the practical benefits of this approach, stating, “By integrating our discussions with bedside learning, we can better understand how to implement what we’ve learned in real-world settings.” (Participant 4, family medicine resident) They believed that this method would enhance the practical application of their knowledge. As one participant noted, “Verifying our team discussion points at the bedside helps bridge the gap between theory and practice, making our learning more effective.” (Participant 30, medical student) The participants realized that medical learning and practice could be enhanced based on bedside learning and discussion. There was a demand for integrated learning at both conferences and bedsides. These insights highlight the importance of combining team discussions with hands-on bedside learning to enhance the practical relevance of their education.

Respecting Diversity and Individual Volition

Participants valued creating a team that respected individual will and thoughts while maintaining team consciousness. One participant stated, “Respecting each team member’s individual will and thoughts is crucial for a cohesive team dynamic.” (Participant 28, family medicine resident) Another participant emphasized the benefits of this approach, noting, “By valuing diverse perspectives, we can enhance our team’s diversity and foster an environment of continuous growth.” (Participant 21, medical teacher) They believed that such respect for individual viewpoints would contribute to a more dynamic and evolving team. As one participant put it, “When we honor each other’s unique contributions, we create a stronger, more innovative team that can adapt and grow together.” (Participant 31, family medicine resident) Through the continual team conferences and discussions, the participants realized that the inclusiveness of the teams could drive their learning and quality of patient care. These insights underscore the importance of balancing respect for individual volition with a unified team consciousness to promote a thriving, diverse organization.

## Discussion

The results of this study emphasize the multifaceted nature of team conferences in rural family medicine departments. Key emerging themes include the need for continuity in patient outcomes, the importance of mutual understanding and individual autonomy, and the enhancement of team healthcare quality through diverse, inclusive learning. These findings align with previous research highlighting the critical role of effective team communication and collaboration in improving patient outcomes and professional development [21,22].

The theme of continuity in patient outcomes reflects the importance of structured and efficient team meetings. Participants expressed concerns over theoretical discussions and the potential disconnect from bedside learning, echoing findings from the previous article, which stressed the need for practical relevance in medical education [23,24]. The connections between medical discussions and practical situations improve learners’ understanding of and motivation for medicine [24]. Besides, time management was a recurring issue, suggesting that carefully structured agendas and timekeeping can enhance the efficiency of these meetings [21]. For practical team conferences, time management as a professional should be respected and demanded.

In rural settings, where healthcare professionals often have multiple responsibilities, the effective management of time is particularly crucial. This study’s participants highlighted the challenge of balancing comprehensive discussions with the practical need for efficient use of time, a concern also noted by the previous article, which found that efficient communication frameworks in healthcare settings significantly improve patient outcomes [25]. This balance is vital in ensuring that team conferences do not detract from direct patient care, which is essential for maintaining high standards of healthcare delivery.

The literature supports the need for psychological safety and respect for individuals in order to enhance team quality. The previous article highlighted that psychological safety is essential for fostering an environment where team members feel comfortable expressing their thoughts and ideas [26]. As this article shows, psychological safety can be generated in rural contexts through continual team conferences and discussions respecting each other. This study’s findings underscore the significance of creating a culture that values each member’s contributions, promoting overall team growth and individual autonomy.

In the context of medical education, the importance of psychological safety cannot be overstated. A supportive environment where trainees feel safe to express concerns and ask questions without fear of judgment is crucial for effective learning. This study found that when participants felt psychologically safe, they were more likely to engage in discussions, leading to more prosperous and productive team conferences. This finding aligns with the previous article, which identified a positive correlation between psychological safety and team learning behaviors [26]. The previous studies in rural contexts suggest that psychological safety can be established in medical education and team management in family medicine [26,27]. Thus, the continuity of team conferences should be enhanced for better psychological safety in rural contexts.

Additionally, respecting individual autonomy while fostering team cohesion was essential. Participants noted that valuing each member’s unique perspectives and experiences enriched discussions and contributed to better patient care. This finding is supported by previous research, which suggests that team diversity can lead to more innovative solutions and improved problem-solving abilities [28]. Diverse ideas and considerations in medical cases can stimulate team members, including medical educators, to learn from each other. Team conferences can become more dynamic and effective by promoting a culture that respects individual differences.

Participants emphasized integrating discussions with bedside learning, a concept supported by Kolb’s experiential learning theory [29]. This integration ensures that theoretical knowledge is effectively applied in clinical practice, improving patient care. The practical application of knowledge discussed during team conferences allows for real-time feedback and adjustments, enhancing the learning experience and patient outcomes. As this article shows, continual discussions in conference rooms and bedsides can motivate learners and improve their learning and relationships among members.

The integration of theoretical and practical learning is critical in medical education. Previous research shows that applying theoretical knowledge in clinical settings solidifies learning and improves clinical skills [29]. This study found that participants who engaged in bedside learning after team conferences could apply the discussed concepts more effectively, leading to better patient management. This approach benefits the residents’ education and ensures patient care remains at the forefront. This approach can drive the idea that the medical team can contribute to better patient care through continual team conferences and discussions on patients’ bedsides.

Additionally, the focus on sustaining participation and motivation aligns with previous articles, which identified intrinsic motivation as a critical factor in maintaining engagement in professional settings [18]. Participants in this study recognized the need for maintaining motivation to ensure continuous participation in team conferences. Identifying individual contributions, providing constructive feedback, and creating a supportive environment were essential for sustaining motivation [30]. Through continual participation, participants in team conferences can realize their contribution to patient care and enhance psychological safety and motivation for team participation.

This study has several limitations. First, the autoethnographic approach, while providing in-depth insights, may introduce bias due to the dual role of the researcher as both a participant and an observer. Future studies could benefit from incorporating external observers to mitigate this bias. Second, the study was conducted in a single rural hospital, which may limit the generalizability of the findings to other settings. Comparative studies across rural and urban healthcare institutions could provide a broader perspective. Third, relying on qualitative data means the findings are context-specific and may not be easily quantified. Future research could include quantitative measures to complement the qualitative insights and provide a more comprehensive analysis.

## Conclusions

This study sheds light on the complex dynamics of team conferences in rural family medicine departments. Effective facilitation, time management, and integrating practical bedside learning with theoretical discussions are crucial for optimizing these meetings. The findings underscore the importance of psychological safety, mutual understanding, and respect for individual differences in enhancing team quality and healthcare outcomes. By addressing these factors, rural healthcare institutions can improve both patient care and the educational experiences of medical professionals. Future research should aim to expand on these findings by incorporating more diverse settings and quantitative measures to validate further and refine the insights gained. This study contributes valuable knowledge to the administration and enhancement of team conferences, ensuring they are both effective in practice and beneficial in educational scope.
